# Association of IL28B Polymorphisms With Hepatitis C Susceptibility in Southern Iran's β‐Thalassemia Population: A Cross‐Sectional Study

**DOI:** 10.1002/hsr2.71648

**Published:** 2025-12-27

**Authors:** Fatemeh Askari, Masoud Kargar, Zeinab Deris Zayeri, Mohammad Ali Jalalifar, Bijan Keikhaei, Gholam Abbas Kaydani

**Affiliations:** ^1^ Department of Laboratory Sciences, School of Allied Medical Sciences Ahvaz Jundishapur University of Medical Sciences Ahvaz Iran; ^2^ Thalassemia and Hemoglobinopathy Research Center, Health Research Institute Ahvaz Jundishapur University of Medical Sciences Ahvaz Iran

**Keywords:** β‐thalassemia, Hepatitis C, IL28B, single nucleotide polymorphism

## Abstract

**Background and Aims:**

Thalassemia is a hereditary hematological condition which interferes with the production of hemoglobin (Hb). Patients with thalassemia require regular blood transfusion, which makes them highly susceptible to pathogens, and especially viral pathogens. Hepatitis C virus (HCV) is also a big threat in that it is hepatotropic in nature and has the potential of causing hepatic injury. Host genetic factors, such as single nucleotide polymorphisms (SNPs) in the IFN λ3 (IL28B) gene, influence the ability to clear the virus and respond to therapy. In this research, we aimed to determine the frequency of the rs8099917 and rs12979860 polymorphisms of the IL28B gene in HCV‐infected thalassemia patients from southern Iran.

**Methods:**

Sixty‐five people with β‐thalassemia were recruited, including 25 people who are HCV positive and 40 people who are HCV negative. The genotyping of the locus at the rs8099917 and rs12979860 sites was done using the tetra‐primer amplification refractory mutation system‐polymerase chain reaction (T‐ARMS‐PCR).

**Results:**

On analyzing the locus at rs8099917, we noted that the TT genotype was common among patients who had HCV, with the proportion of 72% versus 12.5% among HCV‐negative individuals. The GG genotype was also rare in the HCV‐positive group and not present in the HCV‐negative group. On the other hand, TG genotype were found in 20% of the HCV‐positive patients with 87.5% of the HCV‐negative subjects, presenting a very significant difference (*p *< 0.001). On the other hand, the distribution of the rs12979860 genotype was not significantly different in the two groups.

**Conclusion:**

Our findings suggest that the rs8099917 SNP of the IL28B gene may serve as a useful baseline predictor of antiviral responses in thalassemia patients infected with HCV.

**Abbreviations**
ALT: alanine aminotransferase
AST: aspartate aminotransferase
BT: beta‐thalassemia
CHC: chronic hepatitis C
CI: confidence interval
EDTA: ethylenediaminetetraacetic acid
Hb: hemoglobin
HBV: hepatitis virus B
HCC: hepatocellular carcinoma
HCV: hepatitis virus C
HDV: hepatitis virus D
HIV: human immunodeficiency virus
HWE: Hardy‐Weinberg equilibrium
IFN‐λs: interferon lambda
IL28B: interleukin 28B
LD: linkage disequilibrium
NCBI: National Center for Biotechnology Information
OR: odds ratio
PEG‐IFN: pegylated interferon
RBV: ribavirin
SNP: single nucleotide polymorphism
STROBE: strengthening the reporting of observational studies in epidemiology
SVR: sustained virological response
TBE: tris‐borate‐EDTA
T‐ARMS‐PCR: tetra primer‐amplification refractory mutation system‐polymerase chain reaction
WHO: World Health Organization
β‐TM: beta thalassemia major

## 1 Introduction

Thalassemia is defined as a condition characterized by diminished or absent hemoglobin (Hb) production and is recognized as the most common genetic hemoglobin disorder worldwide [1]. Beta thalassemia major (β‐TM) represents a specific form of thalassemia, marked by insufficient production of beta hemoglobin chains, which results in severe anemia during early childhood [2]. According to a 2019 estimate by the World Health Organization (WHO), beta‐thalassemia (BT) affects nearly 50,000 newborns worldwide each year [3]. Within the thalassemia belt, the thalassemia gene is quite prevalent in Iran. It is estimated that there are two to three million carriers of the BT gene, along with 25,000 diagnosed cases of BT in the country [4, 5]. To maintainHb levels between 9 and 11.5 g/dL, managing these patients requires a treatment schedule that includes frequent blood transfusions every 3–4 weeks [6, 7]. The frequency of blood transfusions elevates the risk of transfusion‐mediated hepatitis C virus (HCV) infection in thalassemia patients. The elevated incidence of HCV infection among thalassemia patients in diverse regions further substantiates this susceptibility [8, 9, 10]. The incidence of HCV in thalassemia patients is contingent upon the efficacy of transfusion safety protocols implemented by blood transfusion organizations. Donor screening has significantly mitigated the risk of post‐transfusion infections in affluent environments. Furthermore, the accessibility of safe and effective medications has significantly eliminated HCV in patients who have undergone multiple transfusions [11, 12].

However, in certain resource‐limited settings, the WHO has estimated that up to 25% of blood donations do not undergo screening. Despite strict blood transfusion policies, the thalassemia population in Iran exhibits a high frequency of HCV. This significant prevalence suggests issues with the blood screening methods. Consequently, it is unsurprising that HCV infection remains the most prevalent among patients with thalassemia globally [11, 12].

Interferon lambda (IFN‐λs; IFNL1‐4) is a type III interferon that functions as a cytokine, playing a significant role in generating antiviral immune responses and promoting antitumor activities. It is encoded by the interleukin 28B (IL28B) gene and primarily acts on epithelial surfaces [13, 14]. To date, these genes are the strongest host factors that can predict spontaneous and therapy‐induced clearance of HCV infection. Both genome‐wide and candidate gene studies have demonstrated a connection between IL28B polymorphisms (rs12979860 and rs8099917) and sustained virological response (SVR) to therapy with pegylated interferon and ribavirin (PEG‐IFN/RBV) [15, 16, 17].

The SNP rs12979860 resides within the initial intron of the IFNL4 gene, whereas rs8099917 is positioned 3.6 kb upstream of IFNL4, thereby categorizing it as an IFNL4 variant. The higher expression of type III interferon in individuals with favorable SNP genotypes indicates a potential mechanism connecting these IFNL4 SNPs to SVR [18]. Patients with the TT genotype of rs12979860 or the GG genotype of rs8099917 demonstrate a greater likelihood of positive responses to antiviral therapy for chronic hepatitis C (CHC) [19, 20]. In contrast, CHC patients with unfavorable or risk genotypes for SNPs rs12979860 and rs8099917 face an increased risk of developing hepatocellular carcinoma (HCC) [21]. The advantageous haplotype encompassing both SNPs is significantly correlated with spontaneous viral clearance and reduced hepatic damage, while the detrimental haplotype is linked to hepatic inflammation and fibrosis in individuals with HCV infection. However, there is considerable heterogeneity in IFNL4 genotypes and haplotypes. The prevalence of these advantageous genotypes varies among different ethnic groups; for instance, a higher occurrence of the favorable CC genotype (rs12979860) is observed in CHC patients of European descent compared to African Americans. Furthermore, notable genetic diversity in these IFNL4 haplotypes has been detected in CHC patients from the West Mexico population [22, 23]. Consequently, we aimed to assess the prevalence of the rs8099917 and rs12979860 polymorphisms of the IL28B gene in HCV thalassemia patients within the Iranian population.

## 2 Materials and Methods

### 2.1 Study Design

This cross‐sectional observational study was carried out on 65 patients with thalassemia who were referred to Baghaei 2 Hospital, Ahvaz, Iran, between January 2020 and February 2021.

The research was in compliance with the principles outlined in the Declaration of Helsinki, and it was conducted in line with the STROBE recommendations of conducting observational studies in epidemiology, thus providing clear and standardized reporting. The Research Ethics Committee of Ahvaz University of Medical Sciences gave the required ethical approval (reference number: Th‐9821), and informed consent was obtained in writing before the enrollment of all the participants.

Seventy‐two potential patients were first screened, 65 applicants were selected based on the prespecified inclusion criteria and enrolled. The cohort of study was analytically followed up on 25 cases who were infected with the HCV and 40 non‐infected controls. The inclusion criteria were confirmed thalassemic diagnosis, age of 18 years and above, and availability of a complete clinical and laboratory history. The exclusion criteria included autoimmune or hereditary hepatic disorders (e.g., Wilson disease, hemochromatosis), alcohol consumption over 20 g/day, and co‐infection with hepatitis B virus (HBV), hepatitis D virus (HDV), or human immunodeficiency virus (HIV).

In the infected sub‐group, all subjects showed the presence of detectable HCV RNA at enrolment and were taking a combination therapy with PEG‐IFN/RBV. The non‐infected cohort, in its turn, was not treated with antiviral agents, negative on the HBV, HCV, HDV and HIV serological tests, with regular alanine aminotransferase (ALT) and aspartate aminotransferase (AST) levels.

### 2.2 Sample Collection, DNA Extraction, and Quality Control

Ten millilitres of blood were collected in tubes containing both the anticoagulant ethylenediaminetetraacetic acid (EDTA) and those without it, to facilitate the extraction of viral nucleic acids and host genomic DNA, respectively. Following the manufacturer's recommendations, genomic DNA from the host and viral nucleic acids were extracted from all obtained blood samples using the GeneAll Exgene kit. The DNA samples were assessed for both quantity and quality using the Thermo NanoDrop One (Thermo Fisher, USA).

### 2.3 Molecular Genotyping by T‐ARMS‐PCR, Primers Design, and Gel Electrophoresis

Genotyping of rs8099917 and rs12979860 was performed by tetra‐primeramplification refractory mutation system‐polymerase chain reaction (T‐ARMS‐PCR). The characteristics of primers used for the analyzed gene polymorphisms, such as rs8099917 and rs12979860 are shown in Table 1. The primer's specificity and reliability were validated through BLAST NCBI and positive samples. We used the FlexCycler Thermocycler for the PCR reaction. After adding H_2_O to achieve a final volume of 25 µL, the PCR reaction mixture included 10 µL of 2× master mix (Amplicon, Denmark), 2 µL of DNA (100 ng), 0.3 µL of forward inner primer, 0.2 µL of forward outward primer, 0.3 µL of reverse inner primer, and 0.2 µL of reverse outward primer. The PCR method involved an initial denaturation at 95°C for 5 min, followed by 35 cycles consisting of denaturation at 95°C for 45 s, annealing at 59°C (rs8099917) and 64.5°C (rs12979860) for 40 s, and extension at 72°C for 60 s, with a final extension at 72°C for 3 min. Gel electrophoresis was performed for 45 min using a 1.7% agarose gel in 0.5× tris‐borate‐EDTA (TBE) buffer, running at 100 volts. DNA fragments were visualized using safe stain.

**Table 1:** Sequences of primers used for rs8099917 and rs12979860 polymorphism detection.

SNPs ID	Alleles	Primer sequence (5′–3′)	Allele amplicon size (bp)
rs8099917	T > G	Forward inner primer (T allele): CTTTTGTTTTCCTTTCTGTGAGCAGTT	Product size for T allele: 209
		Reverse inner primer (G allele): TACAGCATGGTTCCAATTTGGGTAAC	Product size for G allele: 283
		Forward outer primer: AACTTCACCATCCTCCTCTCATCC	Product size of two outer primers: 439
		Reverse outer primer: ATAAAGGCTTCTGGTATCAACCCCA	
rs12979860	C > T	Reverse inner primer (C allele): GAGTGCAATTCAACCCTGGTGCG	Product size for C allele: 116
		Forward inner primer (T allele): CCAGGGAGCTCCCCGAAGGAGT	Product size for T allele: 196
		Forward outer primer: CTCTGCACAGTCTGGGATTCCTGGA	Product size of two outer primers: 267
		Reverse outer primer: TCTCCTATGTCAGCGCCCACAATTC	

### 2.4 Data Statistical Analysis

All statistical tests were conducted with the help of the SPSS package of the statistical program, version 26.0 (SPSS Inc., Chicago, IL). GraphPad Prism 8.0 was used in creating the graphical representations. The *χ*² test was used to determine the consistency of genotype distribution through Hardy–Weinberg equilibrium (HWE). The analysis of the normality of the continuous variables was done through the Shapiro–Wilk test prior to the execution of any parametric tests. The comparison between genotype and alleles frequencies between the HCV‐positive and HCV‐negative groups was done using chi‐square and Fisher exact tests as was appropriate. Computations were performed to determine the strength of the relationship between exposure and outcome by obtaining odds ratios (ORs) and 95% confidence intervals (CIs) of these relationships. The comparison of genotypes under the dominant, recessive, codominant and allele models was done using the Fisher's exact test to ensure that the small sample sizes could be accommodated. Haplotype analysis and linkage disequilibrium (LD) mapping were carried out using HAPLOVIEW 4.2 to assess the joint effect of the genetic variants of the candidates of the two studies, that is, the genetic variants of the requirements of both studies.

*χ*² tests were used to compare haplotype frequencies in groups, and D′ and *r*² statistics were used to measure the strength of LD. All statistical tests, including genotype, allele and haplotype comparisons, are pre‐specified and the analysis of data. There was no exploratory or subgroup analysis. All statistical tests had precise *p* values. The a priori level of significance was determined at *α* = 0.05, and all the tests were two‐sided. Statistical methods and reporting were guided by the recommendations of Assel et al. [24] and the SAMPL guidelines [25] for transparent reporting of clinical research. Basic demographic and clinical data were not accessible to all the participants due to the retrospective characteristics of data collection, however, full genotype data were available to all patients, and all analyses performed on full data sets. There were no instances of genotyping failures, and the sample size (*n* =  65) was the same in every analysis.

## 3 Results

### 3.1 Participant Flow

Out of 72 patients who got screened, seven patients were excluded (four patients did not have all the clinical data, and three did not have HCV at all), and 65 patients were included in the ultimate analysis (25 HCV‐positive and 40 HCV‐negative).

### 3.2 Hardy–Weinberg Equilibrium Analysis for rs8099917 and rs12979860 Polymorphisms

HWE was evaluated on both polymorphisms in the case and the control cohort. The HWE of the genotype distributions of rs8099917 and rs12979860 was met with respect to the HCV‐positive participants (*p* > 0.05). On the other hand, non‐observation of HWE existed in both HCV‐negative population and the entire study population of both polymorphisms (see Table 2).

**Table 2:** Hardy–Weinberg equilibrium analysis for rs8099917 and rs12979860 polymorphisms.

Polymorphism	Group	*χ* ^2^	*p* value	Hardy–Weinberg equilibrium
rs8099917	HCV‐positive	2.600047	0.11	Y
	HCV‐negative	24.19753	< 0.001	N
	Total	9.102016	0.003	N
rs12979860	HCV‐positive	2.05585	0.15	Y
	HCV‐negative	6.324195	0.01	N
	Total	8.203287	0.004	N

### 3.3 Descriptive Statistics and Data Availability

All participants did not have access to basic demographic and clinical characteristics, including age, sex distribution, and liver enzyme levels, because the data collection was done retrospectively. Therefore, descriptive statistics could not have been reported in a comprehensive manner. However, overall and confirmed genotype data were available in all the participants, and all inferential tests were carried out on the available data, thus providing the trustworthiness of the associations that were reported. In this regard, the size of the sample that was reported was the same in all the statistical tests.

### 3.4 Frequency of rs8099917 Genotypes and Alleles Among Case and Control Group

**Figure 1:**
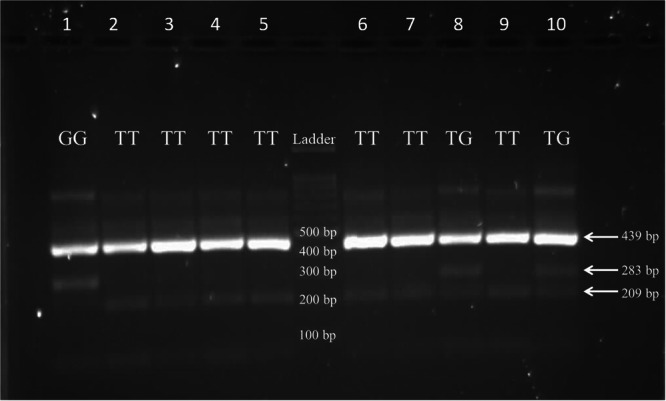
Amplification bands of the different genotypes of SNP rs8099917. The PCR products were separated on a 1.7% agarose gel made in 0.5× TBE buffer by running at 100 V for 45 min and visualized using safe stain. Homozygous TT genotype is identified by a single band at (209 bp) and is observed in wells 2, 3, 4, 5, 6, 7, and 9. Homozygous GG genotype is represented by a single band at (283 bp) and is detected only in well 1. The heterozygous TG genotype also shows a band at (283 bp) and is present in wells 8 and 10. A band at (439 bp) corresponds to the product size amplified by the two outer primers and serves as an internal control for PCR amplification.

In the HCV‐positive group, the TT genotype was most common, and the TG genotype was the most common in the HCV‐negative group. TT (72% vs. 12.5%, *p *< 0.001) and GG (8% vs. 0%) frequencies were significantly higher among HCV‐positive subjects than the HCV‐negative group. On the other hand, the TG genotype was significantly less frequent in the HCV‐positive group (20% vs. 87.5%, *p *< 0.001); compared to the HCV‐negative participants (Table 3). Figure 1 represents the outcomes of gel electrophoresis.

**Table 3:** The frequency of rs8099917 genotypes among case and control groups.

Genotype	Group		Total	*p* value
	HCV‐positive	HCV‐negative		
TT	18 (72%)	5 (12.5%)	23 (35.4%)	< 0.001
TG	5 (20%)	35 (87.5%)	40 (61.5%)	
GG	2 (8%)	0 (0%)	2 (3.1%)	
Total	25 (100%)	40 (100%)	65 (100%)	

There was a significantly higher prevalence of the T allele in the HCV‐positive cohort (82%) than in the HCV‐negative cohort (56.25%). Conversely, HCV‐positive people had a significantly lower frequency of the G allele (18%) compared to those in the HCV negative group (43.75%). Such disparity in the frequencies of alleles was statistically significant (*p *= 0.003, Table 4).

**Table 4:** The frequency of rs8099917 alleles among case and control groups.

Allele	Group		Total	*p* value
	HCV‐positive	HCV‐negative		
T	41 (82%)	45 (56.25%)	86 (66.2%)	0.003
G	9 (18%)	35 (43.75%)	44 (33.8%)	
Total	50 (100%)	80 (100%)	130 (100%)	

### 3.5 Genetic Models of rs8099917 and HCV Infection

In order to examine the correlation between the polymorphism of the rs8099917 and predisposition to HCV infection, a number of genetic models were evaluated (Table 5):

**Table 5:** Association between the various genetic models of rs8099917 and HCV infection.

Genotype	OR (95% CI)	*p* value
GG vs. TT	N/A	> 0.99
TG vs. TT	0.04 (0.01–0.155)	< 0.001
GG vs. TT + TG (recessive model)	N/A	0.14
GG + TG vs. TT (dominant model)	0.056 (0.015–0.2)	< 0.001
TG vs. TT + GG (codominant model)	0.036 (0.009–0.139)	< 0.001
G vs. T (allele model)	0.282 (0.121–0.658)	0.003

Abbreviations: CI, confidence interval; N/A, not available; OR, odds ratio.

GG versus TT: This GG genotype was only identified in HCV‐positive cohort and not in the control. As a result, the OR and the CI were not possible to calculate (OR = N/A, CI = N/A). The exact test of Fisher showed no statistically significant association (*p *> 0.99).

TG versus TT: TG genotype showed a strong protective effect in the face of HCV infection when compared to TT genotype (OR = 0.04, 95% CI = 0.01–0.155, *p *< 0.001).

Recessive model (GG versus TT + TG): Due to the presence of no GG genotype in the control group, OR and CI could not be calculated (OR = N/A, 95% CI = N/A). This association was statistically insignificant (*p *= 0.14).

Dominating model (GG + TG versus TT): Patients who were carrying one G allele (i.e., either TG or GG) had a much lower risk of HCV infection than those with the TT genotype (OR = 0.056, 95% CI = 0.015–0.2, *p *< 0.001).

Codominant model (TG versus TT + GG): TG genotype had a substantial level of protection against the combined TT and GG genotypes (OR = 0.036, 95% CI = 0.009–0.139, *p *< 0.001).

Allele model (G versus T): A G allele was also linked to a significantly lower risk of being infected with HCV (OR = 0.282, 95% CI = 0.121–0.658, *p *= 0.003), which supports its possible protective effect.

### 3.6 Frequency of rs12979860 Genotypes and Alleles Among Case and Control Group

**Figure 2:**
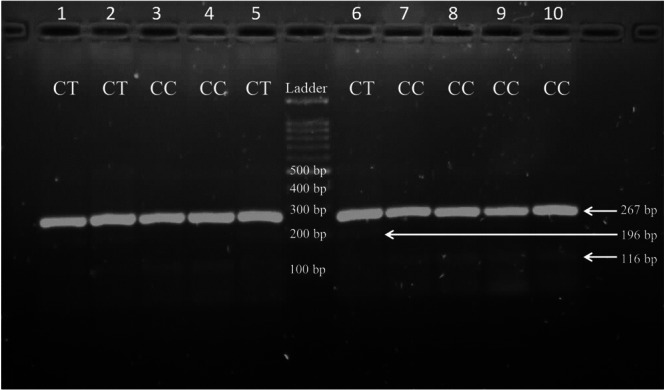
Amplification bands of the different genotypes of SNP rs12979860. The PCR products were separated on a 1.7% agarose gel made in 0.5X TBE buffer by running at 100 V for 45 min and visualized using safe stain. Homozygous CC genotype is identified by a single band at (116 bp) and is observed in wells 3, 4, 7, 8, 9, and 10. The heterozygous CT genotype is represented by a band at (196 bp) and is detected in wells 1, 2, 5, and 6. A band at (267 bp) corresponds to the product size amplified by the two outer primers and serves as an internal control for PCR amplification.

Table 6 illustrates no statistically significant difference in the distribution of the genotypes of the two groups of the genotype of the marker of rs12979860 (*χ* ^2^ test, *p *= 0.85). Figure 2 illustrates the results of the gel electrophoresis. Moreover, the results in Table 7 demonstrate that there are no significant differences between the frequencies of alleles of the rs12979860 in the groups (*χ* ^2^ test, *p *= 0.84).

**Table 6:** The frequency of rs12979860 genotypes among case and control groups.

Genotype	Group		Total	*p* value
	HCV‐positive	HCV‐negative		
CC	10 (40%)	14 (35%)	24 (36.9%)	0.85
CT	14 (56%)	25 (62.5%)	39 (60%)	
TT	1 (4%)	1 (2.5%)	2 (3.1%)	
Total	25 (100%)	40 (100%)	65 (100%)	

**Table 7:** The frequency of rs12979860 alleles among case and control groups.

Allele	Group		Total	*p* value
	HCV‐positive	HCV‐negative		
C	34 (68%)	53 (66.25%)	87 (66.9%)	0.84
T	16 (32%)	27 (33.75%)	43 (33.1%)	
Total	50 (100%)	80 (100%)	130 (100%)	

### 3.7 Genetic Models of rs12979860 and HCV Infection

In order to examine the correlation between the polymorphism of the rs12979860 polymorphism and predisposition to HCV infection, a number of genetic models were evaluated (Table 8):

**Table 8:** Association between the various genetic models of rs12979860 and HCV infection.

Genotype	OR (95% CI)	*p* value
TT vs. CC	1.4 (0.078–25.144)	> 0.99
CT vs. CC	0.784 (0.276–2.223)	0.65
TT vs. CC + CT (recessive model)	1.625 (0.097–27.21)	> 0.99
TT + CT vs. CC (dominant model)	0.808 (0.288–2.264)	0.68
CT vs. TT + CC (codominant model)	0.764 (0.276–2.11)	0.60
T vs. C (allele model)	0.924 (0.435–1.963)	0.84

TT versus CC: The TT genotype showed a slightly higher frequency among HCV‐positive individuals; however, the association was not statistically significant (OR = 1.4, 95% CI = 0.078–25.144, *p *> 0.99).

CT versus CC: The CT genotype was associated with a reduced risk of HCV infection compared to CC, though the difference was not statistically significant (OR = 0.784, 95% CI = 0.276–2.223, *p *= 0.65).

Recessive model (TT vs. CC + CT): The TT genotype showed a higher frequency in the case group, but the association lacked statistical significance (OR = 1.625, 95% CI = 0.097–27.21, *p *> 0.99).

Dominant model (TT + CT vs. CC): The presence of at least one T allele was associated with a lower risk of HCV infection, though not significantly (OR = 0.808, 95% CI = 0.288–2.264, *p *= 0.68).

Codominant model (CT vs. TT + CC): The CT genotype showed a non‐significant protective trend (OR = 0.764, 95% CI = 0.276–2.11, *p *= 0.60).

Allele model (T vs. C): The T allele was slightly more frequent in the control group, but the association with HCV infection was not statistically significant (OR = 0.924, 95% CI = 0.435–1.963, *p *= 0.84).

### 3.8 Haplotype Analysis of rs8099917 and rs12979860

The haplotype analysis was performed to find the joint effect of rs8099917 and rs12979860 polymorphisms on the HCV susceptibility. We used cross‐tabulation and *χ* ^2^ tests to compare haplotype distributions in HCV‐positive and HCV‐negative groups. To evaluate the strength of association for each haplotype, OR and 95% CI were calculated.

The frequency of GC haplotype (G allele of rs8099917 and C allele of rs12979860) was significantly lower in HCV‐positive group as shown in Table 9, suggesting a protective effect. The TT haplotype (T allele of both SNPs) was significantly more frequent in HCV‐positive individuals, which may suggest that it is possibly associated with obtaining the infection.

**Table 9:** Haplotype analysis of rs8099917 and rs12979860 among case and control groups.

Haplotype	Group			*χ* ^2^	*p* value
	HCV‐positive	HCV‐negative	Total		
TC	0.652	0.502	0.56	2.81	0.09
GT	0.152	0.277	0.229	2.723	0.10
GC	0.028	0.161	0.11	5.539	0.02
TT	0.168	0.061	0.102	3.884	0.05

**Figure 3:**
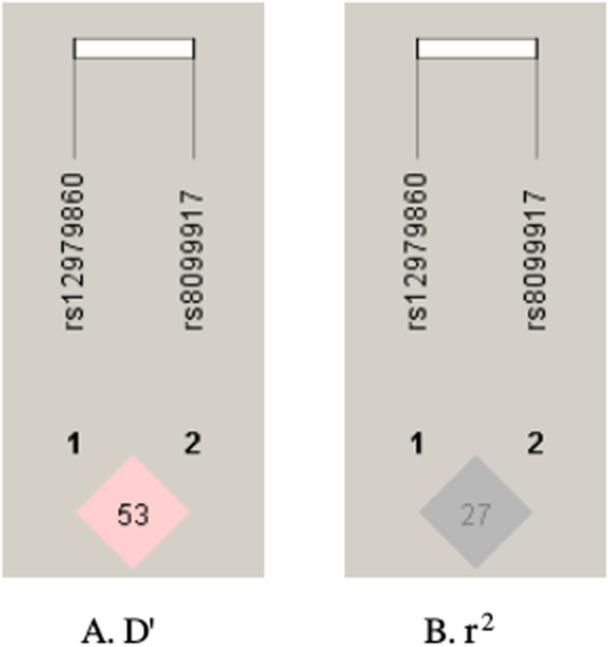
LD block between rs8099917 and rs12979860 SNPs among case and control groups.

Furthermore, a LD analysis was conducted between rs8099917 and rs12979860 to investigate their relationship. The results showed weak LD between the two SNPs in both HCV‐positive and HCV‐negative groups. Table 10 summarizes the results, and Figure 3 shows the results pictorially.

**Table 10:** LD plot analysis of rs8099917 and rs12979860.

D′	LOD	*r* ^2^	95% CI	Distance
0.534	2.72	0.276	0.3–0.68	4378

In summary, the rs8099917 polymorphism, particularly the TG genotype and G allele, showed a significantly protective association against HCV infection under multiple genetic models. On the other hand, rs12979860 was not statistically associated. The GC haplotype showed a protective role, while the TT haplotype may be a risk factor based on haplotype analysis.

## 4 Discussion

This study examined the relationship between IL28B gene polymorphisms (rs8099917 and rs12979860) and HCVinfection among BT patients in the Iranian population. Our results indicated a statistically significant association between the rs8099917 polymorphism and HCV infection status; however, no significant association was found for the rs12979860 polymorphism. These results provide new insights into how host genetic factors may influence susceptibility to HCV infection and possibly affect the effectiveness of antiviral treatment in this high‐risk group. This study concludes that the polymorphism rs8099917 is significantly associated with HCV infection. The TT genotype was found to be significantly more prevalent in the HCV‐positive group (72%) compared to the HCV‐negative group (12.5%), whereas the TG genotype was significantly less prevalent in the HCV‐positive group (20% vs. 87.5%). Importantly, the T allele was also more prevalent in the HCV‐positive group (82%) than the HCV‐negative group (56.25%) (*p *= 0.003). These findings indicate an increase in the inherited punctuations for persistent HCV infection, possibly due to decreased expression of IFN‐λ3 that is critical to antiviral immune responses. On the other hand, TT genotype and T allele of rs8099917 may also be associated with susceptibility to HCV infection, spontaneous viral clearance and SVR among BT patients. This result is consistent with prior reports [26, 27, 28].

In contrast, we found no significant association of rs12979860 polymorphism and HCV infection status in our study (*p *= 0.85 for genotype distribution, *p *= 0.84 for allele frequency); this is contrary to some previous studies indicating a strong association between the CC genotype for rs12979860, or even more generally, IL28B genotypes with favorable outcomes such as SVR to antiviral therapy [27]. Our inability to confirm the association largely reveals issues of sample size (*n* = 65), which reduced power and made it more difficult to detect any differences that may have been significant with a larger sample size, on the one hand, and the genetic variations in IL28B polymorphisms in different populations, on the other hand. The CC genotype for rs12979860 is more common in individuals of European descent compared to those of African‐American or Asian descent, and in an Iranian population, genetic variations that may be specific to certain ethnicities could influence the distribution and significance of rs12979860 genotypes [29].

The analysis of haplotypes showed that the TT haplotype which is composed of the rs8099917 T allele and the rs12979860 T allele was significantly more common in HCV‐positive patients (*p *= 0.05), and the GC haplotype was significantly less common (*p *= 0.02). The results suggest that haplotype combinations could have a significant impact on the risk of HCV infection or the persistence of HCV in individuals with BT. Although, rs8099917 and rs12979860 did have weak LDbetween them in our study population (D′ = 0.534, *r*² = 0.276), rs8099917 and rs12979860 are weakly correlated. This weak LD is consistent with previous reports identifying weak LD between rs8099917 and rs12979860 in other populations [30]. The population‐specific genetic structures demonstrated through weak LD may indicate that population‐specific studies are necessary to dissect the role of IL28B polymorphisms in HCV infection.

Our findings have clinical implications, particularly for patients with BT, who are at higher risk of infection with HCV due to their requirement for frequent blood transfusions. The association of the rs8099917 TT genotype with HCV positivity suggests that this phenotype can be used as a baseline predictor of antiviral therapy response among patients with BT. Patients with the TT phenotype may need special considerations that require more continual monitoring or alternatives to IV or IM PEG‐IFN/RBV therapy, such as DAAs, that have better efficacy [31]. However, cost and access make implementation of monitoring and utilization of alternatives, such as DAAs, challenging in resource‐limited settings, for example, portions of Iran [32]. It is imperative, therefore, to find strategies that will allow suitable improvements to screening and treatment while remaining cost‐effective.

Our research has some limitations. First, we had a small sample size that limited the statistical power to indirectly identify associations, especially rs12979860. Second, our study was conducted at a single center in southern Iran, so our findings may not be generalizable to other countries or populations. Third, we cannot evaluate the potential effects of IL28B polymorphisms on outcomes such as SVR rates because our study was cross‐sectional. Future research is needed with larger sample sizes, multi‐center studies, and longitudinal design. Based on the current research with limited sample sizes, multi‐center studies, and longitudinal designs are required in the future. In addition, the follow‐up of patients with HCV could allow for studies to determine whether the identified IL28B polymorphisms can predict treatment response in patients with BT‐related HCV infection. Additionally, no formal correction for multiple comparisons was applied, which may increase the risk of type I error; therefore, our findings should be interpreted with caution.

## 5 Conclusion

This study demonstrated a clear association with the rs8099917 polymorphism of the IL28B gene and HCV infection in a population of BT patients in the Iranian population. The TT genotype and T allele were significantly more prevalent in the HCV‐positive patients compared to the HCV‐negative patients, thus establishing a potential phenotypic effect of HCV infection in increased susceptibility to the disease and persistent virus or reduced spontaneous viral clearance. The rs12979860 had no significant association, which could possibly be attributed to genetic heterogeneity or the sample size. These findings indicate that genotyping of rs8099917 genetic polymorphism has prognostic utility to identify BT patients at greatest risk for HCV infection, and that may exhibit poor response to antiviral therapy. By facilitating a stratified understanding of risk, in the context of potential treatments such as direct‐acting antivirals, as well as monitoring for persistent infections, IL28B genotyping has the potential to improve clinical understanding in this population. More research should be conducted on larger and multicenter cohorts with respect to rs8099917 and rs12979860, physical mechanisms and how these polymorphisms may play a role in the management of HCV in BT patients.

## Author Contributions

F.A. designed the study. Z.D.Z. contributed to biostatistics analysis. F.A., M.A.J., and G.A.K. contributed to experimental procedure. F.A, B.K., and G.A.K. contributed to experimental analysis. M.K. contributed to manuscript writing, grammar editing, and final version.

## Ethics Statement

Ethical approval was obtained from the Research Ethics Committee of Ahvaz Jundishapur University of Medical Sciences (reference number: Th‐9821).

## Consent

Written informed consent was obtained from all participants prior to enrollment.

## Conflicts of Interest

The authors declare no conflicts of interest.

## Transparency Statement

The corresponding author, Gholam Abbas Kaydani, affirms that this manuscript is an honest, accurate, and transparent account of the study being reported; that no important aspects of the study have been omitted; and that any discrepancies from the study as planned (and, if relevant, registered) have been explained.

## Data Availability

The data sets supporting the findings of this study are available from the corresponding author upon reasonable request.
